# Beyond the Surface, Intravascular Papillary Endothelial Hyperplasia (IPEH): A Case Report

**DOI:** 10.7759/cureus.85268

**Published:** 2025-06-03

**Authors:** Tejraj Kale, Radhika Pathak, Deepa Mane, Punnya Rao

**Affiliations:** 1 Oral and Maxillofacial Surgery, KLE Vishwanath Katti Institute of Dental Sciences, KLE Academy of Higher Education and Research (KAHER), Belagavi, IND; 2 Oral and Maxillofacial Pathology, KLE Vishwanath Katti Institute of Dental Sciences, KLE Academy of Higher Education and Research (KAHER), Belagavi, IND

**Keywords:** benign tumors, intravascular papillary endothelial hyperplasia (ipeh), tongue, tongue mass, tongue neoplasms

## Abstract

This case report aims to contribute to the understanding and documentation of intravascular papillary endothelial hyperplasia (IPEH) in the oral cavity, emphasizing its significance in clinical practice. We report a 65-year-old female patient with reddish swelling over the left lateral border of the tongue for two months. The swelling was non-tender and non-pulsatile. Excisional biopsy under local anaesthesia was done. On histopathological examination, it was diagnosed as IPEH based on two key features: anastomosing and interconnecting papillary projections of endothelial cell proliferation, and the other part of the stroma consisting of a fibrous pseudocapsule comprising the residue of smooth muscles or elastic fibres of blood vessels around the thrombus. Intravascular papillary endothelial hyperplasia constitutes only 2% of all vascular tumours, most commonly of the head and neck region. Since it has no pathognomonic features, it is often misdiagnosed clinically as mucocele, haemangioma, pyogenic granuloma, or melanoma. It is of utmost importance to diagnose this entity correctly, as it is often confused with angiosarcoma both clinically, due to its appearance, and histologically. Complete surgical excision remains the treatment of choice, ensuring definitive management and minimizing the risk of recurrence. IPEH is a rare vascular lesion that poses significant diagnostic challenges due to its clinical and histopathological resemblance to various benign and malignant entities. This case highlights the importance of accurate differentiation, particularly from angiosarcoma, to prevent unnecessary aggressive treatment. A meticulous approach combining clinical assessment, histopathological examination, and patient history is crucial in establishing a precise diagnosis and guiding appropriate management.

## Introduction

Masson’s tumour or Intravascular Papillary Endothelial Hyperplasia (IPEH) is a non-neoplastic, benign, vascular tumour, seen more commonly in the head and neck region [1]. Though common in the head and neck region, it accounts for only 2% of the vascular tumours of the skin and subcutaneous tissues [2]. Clinically, it appears as a bluish-to-reddish, soft-to-firm mass, rarely tender and slow growing, mostly asymptomatic [3,4]. The correct identification and recognition of this lesion is important as it can be misinterpreted both clinically and histopathologically. Clinically, it may be confused with mucocele, hematoma, haemangioma, pyogenic granuloma, nevus, or malignant melanoma. Histologically, it is important to differentiate it from angiosarcoma [4]. The most common sites are the upper lip, lower lip, buccal mucosa, and tongue [5]. Given its rarity and potential for misdiagnosis, documenting cases of IPEH is essential to enhance clinical awareness and diagnostic accuracy. This report presents a unique case of IPEH in the tongue, an uncommon site, highlighting the diagnostic challenges and emphasizing the importance of histopathological confirmation.

## Case presentation

**Figure 1:**
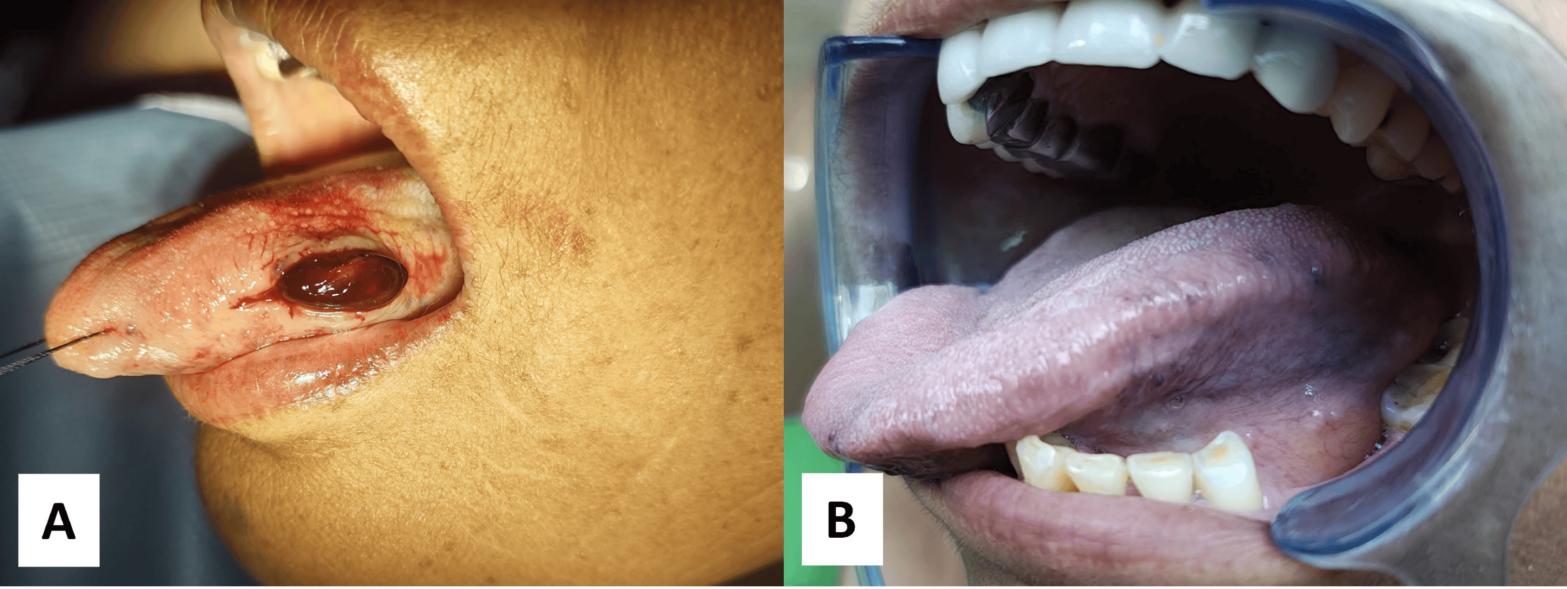
Clinical photographs A: Intraoperative photograph, showing intravascular papillary endothelial hyperplasia (IPEH) of the tongue; B: Postoperative photograph, showing satisfactory wound healing (after 7 days).

A 65-year-old female reported to the Oral and Maxillofacial Surgery OPD with the chief complaint of swelling over the left lateral border of the tongue for two months. The patient gave a history of hysterectomy one year ago. On inspection, the swelling appeared to be reddish in colour with normal overlying mucosa, measuring approximately 1.5 x 1 cm. On palpation, the swelling was soft, fluctuant, non-pulsatile, and non-tender. On examination, the provisional diagnosis was given to be mucocele. But as the swelling was reddish in hue and present on the dorsolateral tongue, which is a rare site for mucocele, the diagnosis of hemangioma was also considered. As the swelling was non-pulsatile, a biopsy was attempted. Excisional biopsy under local anaesthesia was planned; the same was explained to the patient along with the risks and complications. Informed consent was obtained from the patient (Figure 1a).

The patient was administered local anaesthesia. After which, the incision for the excisional biopsy was marked. Excisional biopsy was taken and sent to the lab for histopathological examination. The incision was sutured. The patient was recalled on the seventh postoperative day - wound healing was found to be satisfactory and uneventful. Suture removal was done (Figure 1b).

**Figure 2:**
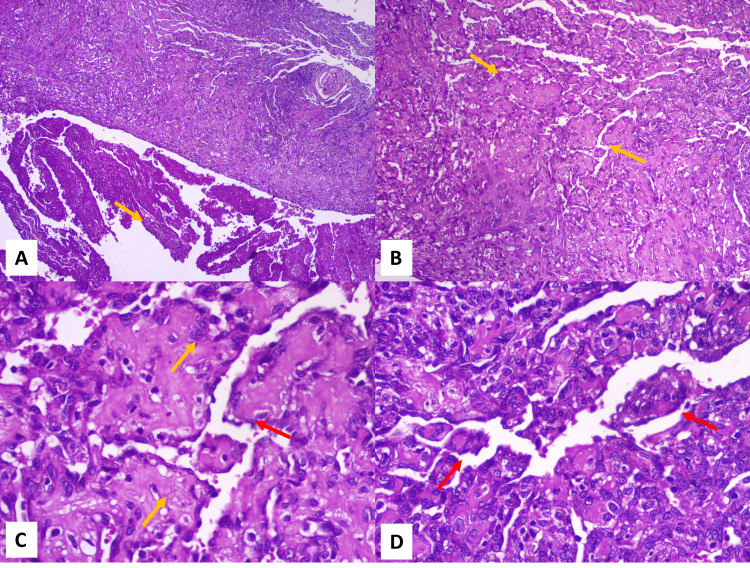
Haematoxylin and eosin-stained sections of the lesion under 4x, 10x, and 40x magnification A: Haematoxylin and eosin-stained section under lower magnification shows endothelial cell proliferation associated with vascular stroma (yellow arrow), and the other part shows parallelly arranged fibres with haemorrhage (4x, H&E). B: Higher magnification shows anastomosing and interconnecting papillary projections of endothelial cell proliferation around numerous small to big blood vessels (yellow arrows) (10x, H&E). C: Endothelial cells appear to be plump, with no evidence of pleomorphism or mitotic figures (yellow arrows). The endothelial cells are seen projecting onto the lumen in a papillary pattern (red arrow) (40x, H&E). D: Higher magnification showing papillary projection (red arrows) of endothelial cells along with surrounding connective tissue (40x, H&E).

Histopathological examination revealed vascular stroma exhibiting endothelial cell proliferation with anastomosing and interconnecting papillary projections around the numerous blood vessels of varying sizes. Hence, it was diagnosed as papillary endothelial hyperplasia with thrombus (Figure 2A-2D).

## Discussion

Intravascular papillary hyperplasia was first described by Pierre Masson in 1923, hence the name “Masson’s tumour”. He had described it as “vegetant intravascular haemangioendothelioma”. The current term used was coined by Clearkin and Enzinger in 1976. The etiopathogenesis of IPEH is not clear. Masson believed the lesion to be neoplastic, Henschen regarded it to be reactive, while some researchers believe it to be an organising thrombus [1,2]. Local trauma/microtrauma to the vessel wall has been associated with the formation of IPEH. But not all cases reported in the literature have been linked to trauma [6]. Some authors believe that intraoral lesions are mostly caused because of local trauma, which leads to dystrophic calcification. The ulcerated or traumatised vascular lesions are repeatedly exposed to calcium ions from the saliva, toothpaste, and dairy products, which leads to organised thrombi with extra calcium salts [7].

Numerous theories have been described for the etiopathogenesis. The neoplastic theory believes that there is a formation of red infarct because of necrosis and degeneration in the vessel lumen caused by endothelial cell proliferation and papillary cell formation. The reactive theory states that perivascular inflammation and blood stasis lead to the proliferation of endothelial cells. Another theory stated it to be a variant of angiolymphoid hyperplasia with eosinophilia, in which there is benign endothelial proliferation that arises from a thrombus [8,9]. Another hypothesis states that in IPEH, there is an increase in the level of basic fibroblast growth factor (bFGF), which is released in response to trauma by macrophages. The bFGF acts as an autocrine factor, leading to an increase in the number of endothelial cells. The endothelial cells also release bFGF, which, because of positive feedback, leads to a cascade of endothelial cell proliferation [4].

Three types of IPEH have been described: the first is the pure type, in which thrombus is associated with characteristically dilated vascular veins. In the second type, preexisting vascular conditions lead to IPEH. In the third type, there is extravascular formation of IPEH [5]. Older patients usually present with the pure type. It is usually associated with veins, not arteries [5]. IPEH does not present with specific clinical characteristics, and hence, it is often confused with other lesions, such as haemangiomas, mucocele, hematoma, and traumatic fibroma [8]. IPEH has no pathognomonic signs and often presents as a nodule or mass that is bluish to reddish in colour, non-tender, and slow-growing. Its diagnosis can only be confirmed after biopsy and histopathological examination. It is more common in females and is usually seen from the fourth to sixth decade of life. Microscopically, it is often confused with angiosarcoma.

IPEH is often well-circumscribed and encapsulated and is usually present intravascularly. If present extra-vascularly, it is often associated with hematomas. No necrotic areas or cellular atypia are present. No invasion of the perivascular space is present. One- or two-cell-thick papillary fronds are present, which are mainly supported by a thrombotic material [10]. However, some authors state that the clinical features of angiosarcoma are also very different from IPEH, as ulceration and necrosis are often clinically evident [1]. A large nuclear protein, Ki-67 (MIB 1), is often absent in the resting phases of the cell cycle. The same protein is present in very small numbers in IPEH, further emphasising the benign and slow-growing nature of the lesion [8]. Complete excision of IPEH remains the treatment of choice. When it occurs with a preexisting vascular lesion, excision of the lesion is the treatment of choice [5]. Recurrence is extremely rare.

## Conclusions

The knowledge about IPEH is extremely important for both the clinician and the histopathologist because of its resemblance to various other lesions. Clinically, its similarity to hemangioma and malignant melanoma can lead to misinterpretation, while histologically, its resemblance to angiosarcoma adds another layer of complexity. A thorough patient history is crucial in distinguishing between these possibilities. Hemangiomas are typically benign vascular proliferations, whereas angiosarcomas are highly aggressive malignant tumors with a poor prognosis. The risk of bleeding during biopsy is an important consideration, especially if the lesion is vascular in nature. Misdiagnosing a benign lesion as angiosarcoma could lead to unnecessarily aggressive treatment, which carries significant morbid consequences for the patient. Conversely, failing to recognize an angiosarcoma early could delay life-saving intervention. Hence, a meticulous approach to diagnosis, incorporating clinical evaluation, histopathological analysis, and patient history, is essential to ensure accurate identification and appropriate management of the lesion, preventing unnecessary interventions or misdiagnoses with serious consequences.
